# Abnormal Fractional Amplitude of Low Frequency Fluctuation Changes in Patients With Dry Eye Disease: A Functional Magnetic Resonance Imaging Study

**DOI:** 10.3389/fnhum.2022.900409

**Published:** 2022-05-24

**Authors:** Rong-Bin Liang, Li-Qi Liu, Wen-Qing Shi, Tie Sun, Qian-Min Ge, Qiu-Yu Li, Hui-Ye Shu, Li-Juan Zhang, Yi Shao

**Affiliations:** Department of Ophthalmology, The First Affiliated Hospital of Nanchang University, Jiangxi Center of National Ocular Disease Clinical Research, Nanchang, China

**Keywords:** dry eye, fractional amplitude of low frequency fluctuation, anxiety, depression, brain function

## Abstract

**Purpose:**

To investigate spontaneous brain activity in patients with dry eye (DE) and healthy control (HC) using the fractional amplitude of low frequency fluctuation (fALFF) technique with the aim of elucidating the relationship between the clinical symptoms of DE and changes in brain function.

**Material and Methods:**

A total of 28 patients with DE and 28 matched healthy volunteers (10 males and 18 females in each group) were enrolled. Resting-state functional magnetic resonance imaging scans were performed in both groups. Then all subjects were required to complete a comprehensive Hospital Anxiety and Depression Scale (HADS). Receiver operating characteristic (ROC) curve analysis was used to evaluate the differences in fALFF values between the two groups and their diagnostic value. Linear correlations between HADS and fALFF values in different brain regions of DE patients were analyzed using the *Pearson* correlation coefficient.

**Results:**

Patients with DE had significantly higher fALFF values in the left calcarine sulcus (CS) than the HC group, while fALFF values in the bilateral middle frontal gyrus (MFG) and right MFG/right inferior frontal gyrus (IFG) were significantly lower in DE patients than in HC group. fALFF values had a high diagnostic value for differentiating patients with DE from the HC group (*P* < 0.001). Right MFG and right MFG/IFG were significantly correlated with HADS values.

**Conclusion:**

Our study found that DE mainly involved functional disorders in the brain areas of the left CS, bilateral MFG and right MFG/right IFG, which helped us to find possible clinical features of DE disease and reflected the potential pathological mechanism of DE.

## Introduction

Dry eye (DE) is a multifactorial chronic ocular surface disorder commonly encountered in ophthalmology. The prevalence of DE ranges from 5% to 50% and can be as high as 75% in adults over the age of 40, with women being one of the most commonly affected groups (Stapleton et al., 2017). The etiology of DE is complex and the causative mechanisms are not yet fully elucidated. Common risk factors include advanced age, female sex, low humidity environment, systemic medication, and autoimmune diseases. The prevalence of DE is higher in people working at video terminals and in those exposed to harsh environments, such as high altitude, low air pressure, hypoxia, wind, and strong ultraviolet light, for long periods of time. Diabetes, keratoconus surgery, pterygium, allergic conjunctivitis, dry syndrome, and ocular surface drug abuse are strong risk factors for the development of DE (Jiang et al., 2017). DE can seriously affect the ability to perform daily activities, such as reading, using a computer, and driving, resulting in reduced quality of life and increased social burden.

Individuals with DE are more likely than those without the condition to have a visual impairment, neuropathic pain, anxiety, depression, and other symptoms (Weatherby et al., 2019). The mechanism underlying the development of these symptoms and whether they are related to brain dysfunction is not fully understood. Hence, it is important to explore whether abnormal changes occur in the brains of patients with DE relative to healthy controls.

Functional magnetic resonance imaging (fMRI) is a very mainstream, non-invasive, and safe imaging technique that is commonly used to detect some brain diseases and neurological abnormalities (Khosla et al., 2019). We can use fMRI to examine the functional metabolism of the brain in DE patients to further understand the mechanisms of abnormal brain function in DE patients. In recent years, more and more scholars have studied the abnormal alterations in the brain of DE, and the study of DE from central nervous system functionalism is called for, thus using fMRI technology provides us with an effective means to explore the central changes in DE patients. Yan et al. (2020) detected abnormal limbic-cortical circuit ReHo values in patients with DE using resting-state MRI. Many past researches have shown that in patients with Sjögren’s syndrome using fMRI, abnormalities in brain function have been found in the white matter (Lauvsnes et al., 2014), cerebellar gray matter (Tzarouchi et al., 2011), the hippocampal region (Zhang et al., 2020), and the frontoparietal and visual cortex regions (Xing et al., 2018). Until now, there are few studies on the changes in brain function in patients with DE, Resting-state MRI can noninvasively assess the functional status of the brain at the visual cortex and visual pathway levels. Therefore, in this study, we used fractional amplitude of low frequency fluctuation (fALFF) in patients with severe dry eye to explore changes in the functional activity of various brain regions in patients with DE, with a view to further understanding the neuropathological mechanisms of DE.

## Materials and Methods

### Subjects

**Figure 1:**
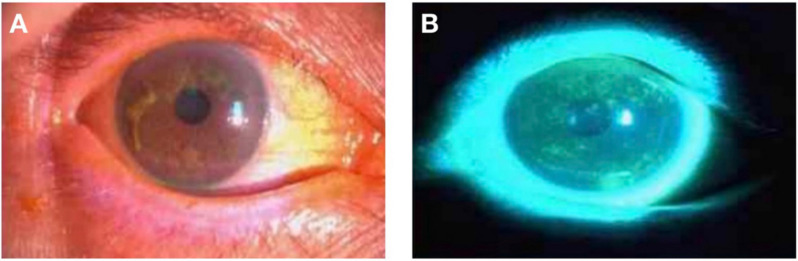
Photographs of the anterior segment of a DE patient taken using slit- lamp microscopy. Panels **(A,B)** are photographs of the preocular segment of the same patient under slit lamp microscopy, and **(B)** is a photograph after sodium fluorescein staining. Abbreviations: DE, dry eye.

We recruited patients with DE (*N* = 28; 18 females and 10 males) who attended the Ophthalmology Department of the First Affiliated Hospital of Nanchang University. The diagnostic criteria for DE were as follows: (1) patients complained of one of the subjective symptoms, including ocular dryness, foreign body sensation, burning sensation, fatigue, discomfort, blurred vision, or eye pain; and (2) a score of ≥7 on the Chinese Dry Eye Questionnaire scale or an Ocular Surface Disease Index (OSDI) score of ≥13; and tear film rupture time <5 s or Schirmer I test (without anesthesia) ≤5 mm/5 min (Figure 1).

Inclusion criteria for patients with DE were as follows: (1) met the diagnostic criteria for DE; and (2) were between 40 and 60 years of age. Exclusion criteria were as follows: (1) ocular anomalies, such as conjunctival scarring, atresia of the lacrimal gland opening, or complete atrophy of accessory lacrimal glands; (2) other ocular diseases associated with other conjunctiva, cornea, or iris; (3) pregnant or lactating women; (4) suspected or confirmed history of substance abuse; (5) systemic systemic diseases such as Sjogren’s syndrome, Stephens-Johnson syndrome, systemic lupus erythematosus; (6) presence of metallic foreign bodies in the body, such as pacemakers; (7) suffering from a neurological or psychological disorder; and (8) factors that may affect the study results, such as a history of alcohol consumption or smoking, claustrophobia, etc.

We also recruited 28 healthy volunteers as the HC group (18 females and 10 males) who were matched to DE patients in terms of age, gender, and other demographic parameters. HC group met the following criteria: (1) no brain parenchymal abnormalities on MRI; (2) no other ocular diseases and corrected visual acuity; (3) no abnormalities on neurological examination; and (4) no contraindications to MRI.

All study procedures followed the Declaration of Helsinki, were in accordance with the principles of medical ethics, and were approved by the Medical Ethics Committee of the First Affiliated Hospital of Nanchang University. All subjects signed the relevant informed consent forms before the examination and were informed of the study objectives, methods, and associated risks.

### Hospital Anxiety and Depression Scale (HADS) Score Acquisition

All subjects were assessed by the same professionally trained and highly experienced psychological nurse. The first step was to ensure that the assessment was completed independently, with calm mental activity, and in the most realistic way possible. If the subject had a literacy impairment, the nurse completed it for them by describing the meaning of the questions in detail. The HADS scale consists of a total of 14 questions, including two subscales consisting of anxiety (Group A questions) and depression (Group D questions). The values in front of the four options represent the final score for the question change (Jones et al., 2017).

### MRI Data Acquisition

MRI was performed with a 3-Tesla magnetic resonance scanner (Magnetom Trio; Siemens, Munich, Germany). Subjects were instructed to keep their eyes closed but remain awake and relaxed until the end of the scan. Data were obtained with a 3D spoiled gradient-recalled echo sequence. The imaging parameters of the T1-weighted and T2-weighted image sequences (176 images) were as follows: repetition time (TR) = 1,900 ms, echo time (TE) = 2.26 ms, thickness = 1.0 mm, gap = 0.5 mm, acquisition matrix = 256 × 256, the field of view = 250 × 250 mm, and flip angle = 9°. Imaging parameters for the 240 functional images were as follows: *TR* = 2,000 ms, *TE* = 30 ms, thickness = 4.0 mm, gap = 1.2 mm, acquisition matrix = 64 × 64, flip angle = 90°, field of view = 220 × 220 mm, and 29 axials. Scanning times were 5 and 10 min, respectively.

### fALFF Analysis

The fALFF value was calculated on the trend data by REST software. REST had a built-in fast Fourier transform function that converts time series data into the frequency domain and calculates the power spectrum. Using the ratio of each frequency in the low-frequency range (0.01–0.08 Hz) to the power in the whole frequency range (0–0.25 Hz), fALFF was obtained.

### Functional Magnetic Resonance Data Analysis

The fALFF values of the DE and HC group were made more normally distributed by Fisher *Z*-transformation. ThefALFF values were analyzed by independent two-sample *t*-test using SPM8-based REST software, and the False Discovery Rate (FDR) correction method was used to perform multiple testing hypothesis corrections. The width at half height (FWHM) was set to 4 mm × 4 mm × 4 mm and set *Q* < 0.01.

### Correlation Analysis

All patients were asked to complete the HADS, and the resulting data were analyzed for correlation with fALFF values using GraphPad Prism8 (GraphPad Software, Inc. La Jolla, CA, USA), and correlation graphs generated based on the results.

### Statistical Analysis

We used SPSS 22.0 (SPSS, IBM Corporation, USA) software to perform independent sample *t*-tests and chi-squared tests to determine the differences between the data of this experiment. The 2-sample *t*-test was applied to determine the difference in mean fALFF values between DE patients and the HC group; receiver operating characteristic (ROC) curves were used to determine the diagnostic value of fALFF values in each brain region for DE patients. *Pearson* correlation coefficient analysis was applied to explore the relationship between the mean fALFF values of each brain region and their clinical behaviors in the DE group. In all our statistical analyses, *p*-values <0.05 considered that the differences were statistically significant.

## Results

### Demographic Information and Visual Measurements

There were no significant differences in age (*p* = 0.674), weight (*p* = 0.628), height (*p* = 0.138), body mass index (*p* = 0.111), or years of education (*p* = 0.940) between patients with DE and HC (Table 1).

**Table 1:** Clinical characteristics of patients between DE and HC group.

Characteristics	DE	HC	*t*-value	*p*-values
Male/female	10∖18	10∖18	NA	NA
Age (years)	49.07 ± 4.45	48.78 ± 4.25	0.423	0.674
Weight (kg)	58.29 ± 3.65	58.75 ± 3.33	−0.488	0.628
Height (cm)	167.50 ± 7.07	164.93 ± 5.38	1.504	0.138
BMI (kg/m^2^)	20.87 ± 2.03	21.57 ± 1.34	−1.619	0.111
Years of education (years)	10.29 ± 1.61	10.25 ± 1.90	0.076	0.940

*Notes: Independent t-tests comparing the two groups (*P* < 0.05) represented statistically significant differences). Abbreviation: DE, dry eye; HC, healthy control; NA, not applicable; BMI, body mass index*.

### Differences in fALFF Values Between Patients With DE and HC

**Figure 2:**
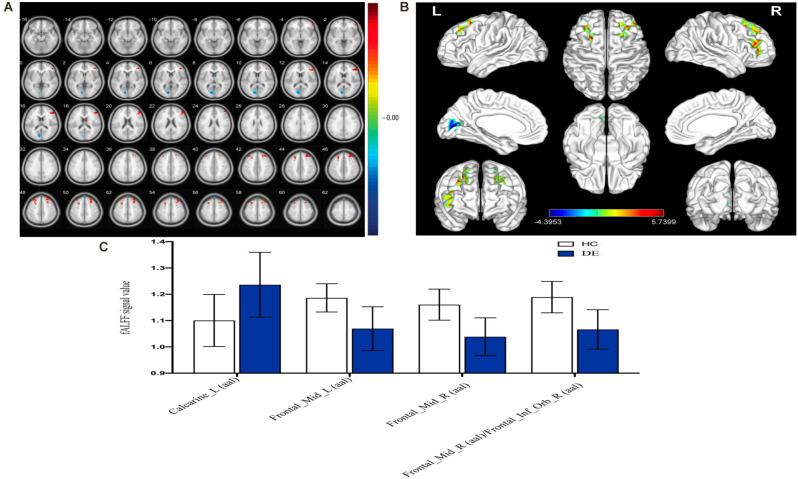
Spontaneousbrain activity in the DE and HC groups. **(A)** The different ALFF regions between the DE and HC group. **(B)** Significant difference in brain activity in the cerebrum. The red regions indicate higher ALFF values, and the blue regions imply lower AFLL values. **(C)** The mean ALFF values between DE and HC groups. Abbreviations: fALFF, fractional amplitude of low-frequency fluctuation; L, left; R, right. Calcarine_L (aal), calcarine sulcus; Frontal_Mid_L (aal), left middle frontal gyrus; Frontal_Mid_R (aal), right middle frontal gyrus; Frontal_Mid_R (aal)/Frontal_Inf_Orb_R (aal), right middle frontal gyrus/right inferior frontal gyrus.

As shown in Figure 2 and Table 2, the fALFF values in the left calcarine sulcus (CS) were significantly higher in patients with DE than in the HC group, whereas fALFF values in the bilateral middle frontal gyrus (MFG) and right MFG/right inferior frontal gyrus (IFG) were significantly lower in patients with DE than in the HC group.

**Table 2:** Brain regions with significant differences in fALFF values between DE and HC group.

Brain areas	MNI coordinates			Number of voxels	*T*-value
	X	Y	Z		
HC>DE					
Frontal_Mid_R (aal)/Frontal_Inf_Orb_R (aal)	51	36	15	93	5.7399
Frontal_Mid_R (aal)	30	39	45	99	5.6994
Frontal_Mid_L (aal)	−24	18	57	65	4.7412
HC<DE					
Calcarine_L (aal)/brodmann area 17/brodmann area 18	−6	−81	15	86	−4.3953

*Note: The statistical threshold was set at the voxel level with *P* < 0.05 for multiple comparisons using False Discovery Rate (*Q* < 0.01 and cluster size >15). Abbreviations: fALFF, fractional amplitude of low-frequency fluctuation; L, left; DE, dry eye; HC, healthy control; MNI, Montreal Neurological Institute; R, right; Calcarine_L (aal), calcarine sulcus; FrontalMidL (aal), left middle frontal gyrus; FrontalMidR (aal), right middle frontal gyrus; FrontalMidR (aal)/FrontalInfOrbR (aal), right middle frontal gyrus/right inferior frontal gyrus*.

### ROC Curve Analysis

**Figure 3:**
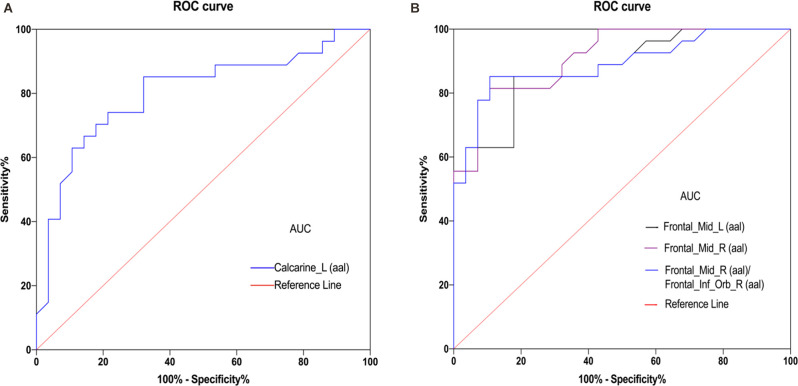
ROC curve analysis of the mean ALFF values for altered brain regions. **(A)** The area under the ROC curve was 0.8022 (*P* < 0.0001; 95% confidence interval (CI) = 0.681–0.924) for Calcarine_L (aal). **(B)** The area under the ROC curve was 0.8763 (*P* < 0.0001; 95% CI = 0.786–0.966) for Frontal_Mid_L (aal); Frontal_Mid_R (aal) 0.9134 (*P* < 0.0001; 95% CI = 0. 842–0.985); Frontal_Mid_R (aal)/Frontal_Inf_Orb_R (aal) 0.8909 (*P* < 0.001; 95% CI = 0. 802–0.980). Abbreviations: fALFF, fractional amplitude of low-frequency fluctuation; AUC, area under the curve; ROC, receiver operating characteristic; L, left; R, right; Calcarine_L (aal), calcarine sulcus; Frontal_Mid_L (aal), left middle frontal gyrus; Frontal_Mid_R (aal), right middle frontal gyrus; Frontal_Mid_R (aal)/Frontal_Inf_Orb_R (aal), right middle frontal gyrus/right inferior frontal gyrus.

Given that fMRI can detect abnormal activity in certain brain regions, we investigated the diagnostic value of fALFF values in patients with DE by ROC curve analysis. As shown in Figure 3, the AUC value for the left CS was 0.8022 (95% confidence interval(CI): 0.681–0.924; *p* < 0.0001), that for the left MFG was 0.8763 (95% CI: 0.786–0.966; *p* < 0.0001), that for the right MFG was 0.9134 (95% CI: 0.842–0.985; *p* < 0.0001), and the AUC value of the right MFG/right IFG was 0.8909 (95% CI: 0.802–0.980; *p* < 0.0001). These results suggest that fALFF values in these brain regions exhibit good accuracy and are valuable for differentiating patients with DE from HC.

### Correlation Analysis

**Figure 4:**
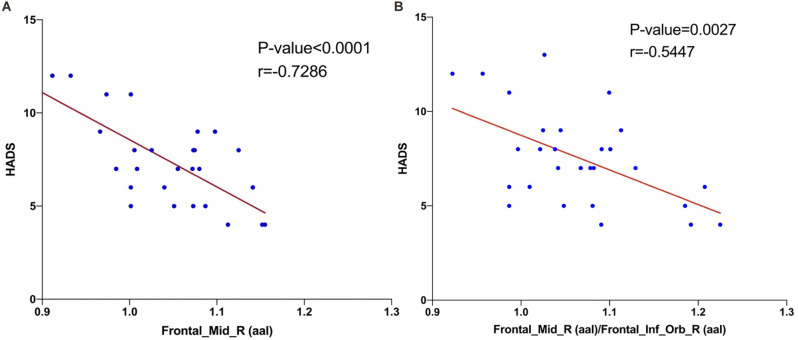
Correlations between the HADS and ALFF values of brain area. The results shown in **(A)** and **(B)** both represent a negative correlation with the HADS and fALFF values in right middle frontal gyrus (*r* = −0.7286, *P* < 0.0001) and right middle frontal gyrus/right inferior frontal gyrus (*r* = −0.5447, *P* = 0.0027), respectively. Abbreviations: HADS, Hospital Anxiety and Depression Scale; fALFF, fractional amplitude of low-frequency fluctuation.

The results of *Pearson* correlation coefficient analysis in Figure 4 and Table 3 showed that fALFF values in right MFG (*r* = −0.7286, *P* < 0.0001) and right MFG/right IFG (*r* = −0.5447, *P* = 0.0027) were significantly and negatively correlated with HADS values.

**Table 3:** Pearson correlations analysis.

Brain regions	fALFF value (mean ± SD)	HADS (mean ± SD)	*r*-value	*P*-value
Calcarine_L (aal)	1.2363 ± 0.1232	7.5357 ± 2.4854	−0.2641	0.1744
Frontal_Mid_L (aal)	1.0694 ± 0.0831		−0.3661	0.0553
Frontal_Mid_R (aal)	1.0384 ± 0.0720		−0.7286	<0.0001
Frontal_Mid_R (aal)/Frontal_Inf_Orb_R (aal)	1.0663 ± 0.0750		−0.5447	0.0027

*Note: fALFF, fractional amplitude of low-frequency fluctuation; SD, standard deviation*.

## Discussion

The global prevalence of DE is estimated to be 5%–50%, with a prevalence of approximately 5.7% in women under 50 years of age, increasing to 9.8% in postmenopausal women (Verjee et al., 2020). DE can cause eye discomfort, visual disturbances, neuropathic pain, anxiety, and depression, among other symptoms. Further, DE has a long chronic onset and can progress without intervention, seriously affecting the quality of life and increasing social burden (Matossian et al., 2019). To date, although fALFF has been studied in neurological disorders, very few studies have applied fALFF in the investigation of DE. The fMRI is an emerging imaging technique that is widely used in the study of neurological diseases. Currently, fMRI has been used in a variety of ophthalmic and neurological diseases (Table 4). The purpose of this study was to explore the changes in spontaneous brain activity in patients with DE using fALFF, with the aim of providing new evidence regarding the neuropathological mechanisms underlying the condition and its diagnosis.

**Table 4:** Application of fALFF in ophthalmology and other diseases.

Author	Year	Disease	References
**Ophthalmological diseases**			
Wen-Feng Liu et al.	2019	Exophthalmos of Primary Hyperthyroidism	Liu et al. (2019)
Rong Wang et al.	2021	Primary angle-closure glaucoma	Wu et al. (2020)
Zhu F et al.	2019	Corneal ulcer	Zhu et al. (2019)
Shi WQ et al.	2019	Monocular blindness	Shi et al. (2019)
**Neurogenic diseases**			
Natalia Egorova et al.	2017	Post-stroke	Egorova et al. (2017)
Liu Yang et al.	2018	Alzheimer Spectrum	Yang et al. (2018)
Anny Reyes et al.	2016	Epilepsy	Reyes et al. (2016)



**Figure 5:**
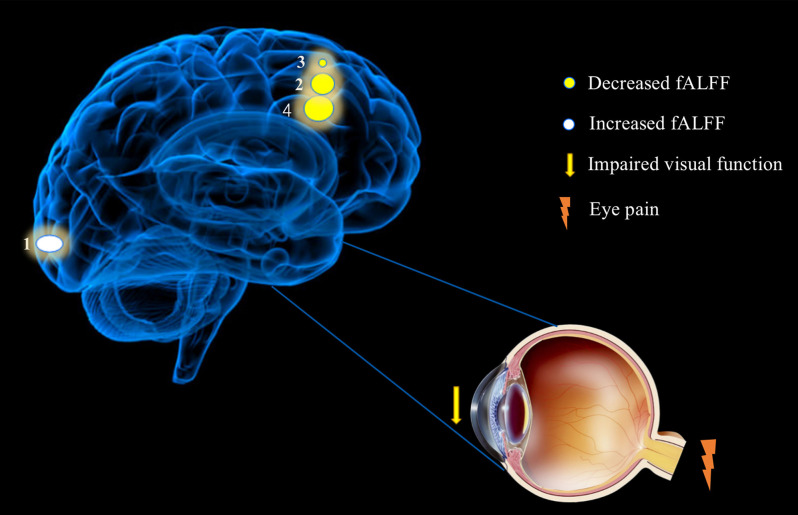
Significant differences in spontaneous brain activity between the DE and HC groups. Different brain regions that were observed: (1) Calcarine_L (aal); (2) Frontal_Mid_R(aal); (3) Frontal_Mid_L(aal); (4) and Frontal_Mid_R(aal)/Frontal_Inf_Orb_R (aal). The white areas present brain regions with increased fALFF values and the yellow areas are brain regions with decreased fALFF values. Abbreviations: fALFF, fractional amplitude of low-frequency fluctuation; L, left; R, right; Calcarine_L (aal), calcarine sulcus; Frontal_Mid_L (aal), left middle frontal gyrus; Frontal_Mid_R (aal), right middle frontal gyrus; Frontal_Mid_R (aal)/Frontal_Inf_Orb_R (aal), right middle frontal gyrus/right inferior frontal gyrus.

Compared with the HC group, patients with DE had significantly higher fALFF values in the left CS and significantly lower fALFF values in the bilateral MFG and right MFG/right IFG. Figure 5 shows the abnormal brain regions.

### Analysis of Elevated fALFF Values in Patients With DE

The occipital lobe is a relatively small part of the posterior end of the cerebral hemisphere, containing the visual cortex and primary visual areas. In humans, the primary visual cortex (V1) is mainly located in the occipital cortex (Brodmann area 17/Brodmann area 18) above and below the CS, which receives impulses from the upper retina, below the CS, which receives impulses from the lower retina, and below the posterior lower 1/3 of the CS, which receives impulses from the macula (De Moraes, 2013). V1 receives visual information from the lateral geniculate body and is responsible for the perception of natural scenes and color signals (Weliky et al., 2003). Impaired optic nerve function can lead to dysfunction of the visual cortex, and the connections between neurons in the visual cortex are reduced in blind people (Fox and Raichle, 2007). In a study by Williamson et al. (1992), patients with abnormal occipital lobe discharges were found to experience hallucinations, episodic blackouts, abnormal eye movements, visual field defects, and other disorders. Further, a study by Rossion et al. (2003) determined that damage to the occipital lobe can result in reduced recognition of faces, and abnormalities in the functional connectivity of primary visual cortical areas have been reported in studies of refractive parametric amblyopia (Ding et al., 2013), strabismus (Yan et al., 2019), and glaucoma (Wang et al., 2017). In the present study, fALFF values from the left CS were significantly increased in patients with DE compared with the HC group, suggesting that activity of this brain region is enhanced in patients with DE, which improves visual information processing. As DE can cause damage to patients’ vision, we speculate that the increase in fALFF values in the CS visual cortex may be a compensatory result for the visual damage caused by DE.

### Analysis of Reduced fALFF Values in Patients With DE

The MFG is located in the anterior part of the brain and is responsible for language processing, stress response (Carter et al., 2006), cognitive function (Achiron et al., 2013), and attention control (Japee et al., 2015). Many disorders are associated with MFG dysfunction; for example, attention deficit hyperactivity disorder (ADHD; Tafazoli et al., 2013) and schizophrenia (Quan et al., 2013). Patients with depression also show significant MFG dysfunction (Andersson et al., 2009). The MFG is involved in the regulation of downstream nociception, and dysfunction of this circuit can lead to pain (Mylius et al., 2006); for example, reduced gray matter in the MFG was found in patients with cluster headaches (Absinta et al., 2012). Sprenger et al. (2007) used [18F]-fluorodeoxyglucose positron emission tomography to measure significantly reduced frontal lobe glucose metabolism in patients with cluster headache, which indicated dysfunction in pain modulation regions. In the present study, we found that the fALFF values from bilateral MFG were significantly lower in patients with DE, suggesting abnormal MFG function. Therefore, we hypothesize that MFG dysfunction may be associated with the occurrence of ocular pain, anxiety, and depression in patients with DE.

The IFG is closely related to emotional (Tabei, 2015) and attention (Ochsner et al., 2012) regulation and is also involved in the protection of memory (Lin et al., 2017). In children with autism spectrum disorders, sleep deprivation is associated with an increase in negative affect and a decrease in positive affect and psychomotor performance, and brain function analysis revealed alterations in several brain regions, including a decrease in the connection between the IFG and the thalamus (Li et al., 2021). Further, IFG white matter connectivity was significantly reduced in patients with compulsive disorder (Goncalves et al., 2015). Coordination between vision and movement relies on the frontoparietal network, which receives visual and proprioceptive inputs, and the IFG is involved in the integrated processing of visual-motor sensory information (Papadelis et al., 2016). In terms of molecular mechanisms, IFG γ-aminobutyric acid type A receptor binding affinity is associated with cognitive impairment and γ-aminobutyric acid type A receptor binding affinity is positively associated with sustained visual attention (Kasagi et al., 2018). In the present study, we found that the fALFF values from the IFG were significantly lower in patients with DE than in the HC group, which may be related to the negative emotions associated with visual impairment and chronic ocular discomfort in patients with DE.

Numerous previous studies have reported clear correlations between dry eyes and anxiety and depression, where patients with DE have reduced tear film stability, intense ocular discomfort, and greater susceptibility to adverse mood (van der Vaart et al., 2015). A population-based cross-sectional study in Beijing, China that scored depression in 1,456 patients with DE found that depression was markedly more prevalent in patients with DE than in those without (Labbe et al., 2013). In a study of patients with moderate to severe depression, cognitive deficits in depressed patients were associated with primary visual cortex function at the CS (Desseilles et al., 2011). A voxel-based morphometric study found significant reductions in gray matter volume in the MFG, IFG, and middle temporal gyrus regions in patients with major depression (Kandilarova et al., 2019). In a study based on regional homogeneity (ReHo), depressed patients had lower ReHo in the right MFG and left precentral gyrus (Geng et al., 2019). IFG is an indirect inhibitor of the amygdala, and a study of 137 anxious patients found significantly impaired IFG function, which was associated with a weaker reciprocal excitatory connection between the IFG and vmPFC (Cha et al., 2016). The reality of our findings (Figure 4) is that the fALFF value of right MFG (*r* = −0.7286, *P* < 0.0001) and the right MFG/right IFG value (*r* = −0.5447, *P* = 0.0027) were significantly and negatively correlated with the HADS value. This suggests a potential association between the changes in brain function occurring in DE patients and their greater susceptibility to anxiety and depression.

In our study, we found that the fALFF values in the vicinity of the CS of patients with DE were significantly higher than those in the HC group, while the fALFF values at the MFG and IFG in patients with DE were significantly lower than those in the HC group. Visual impairment, chronic neuropathic pain, ocular discomfort, and reduced emotional regulation caused by brain dysfunction in patients with DE are associated with neuropathy mechanisms underlying anxiety and depression (Bai et al., 2020).

**Figure 6:**
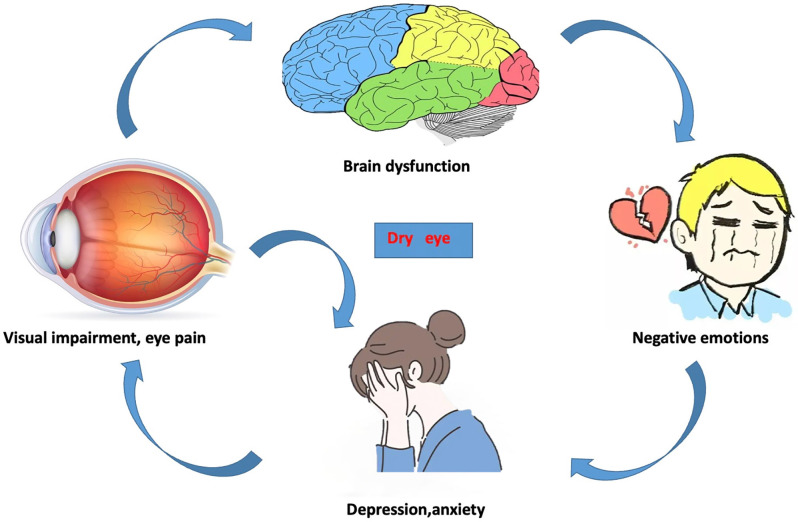
The relationship between changes in internal eye states and vision, brain activity, and emotional state.

In conclusion, long-term DE brings negative emotions and reduces the quality of life of patients, we found that the fALFF values of CS, MFG, and IFG of DE patients are abnormal compared with those of the HC group by resting-state MRI.ROC curve analysis of left CS, left MFG, right MFG and right MFG/right IFG involved in this study have high differential diagnosis significance indicating that the fALFF values of MFG and IFG combined can be used as a potential imaging biomarker to discriminate between DE and HC groups. We speculate that DE may cause brain dysfunction (Table 5, Figure 6). The neurological basis for the pathophysiology of the dry eye provided by the present study may lead to a new field of research to define this basis more precisely with the aim of providing a new idea for the diagnosis and treatment of patients with DE.

**Table 5:** Brain regions alternation and its potential impact.

Brain regions	Experimental	Brain function	Anticipated results
Calcarine sulcus	DE>HC	Natural scene coding Color signal perception.	Visual impairment, identify obstacles, etc.
Middle frontal gyrus	DE<HC	Processing of language cognitive function, and Pain perception.	Depression, headaches, poor concentration, etc.
Inferior frontal gyrus	DE<HC	Visual processing, emotional processing.	Visual impairment, dementia, etc.

*Note: DE, dry eye; HC, healthy control*.

There are some limitations to this study; first, our study sample size was relatively small, which may directly lead to less reliable results, and in order to validate our findings, we need to conduct the study in a larger population. Then, we did not group the degree of disease of DE, and at a later stage, we need to further group the severity of DE on the basis of a large sample for comparison.

## Data Availability Statement

The original contributions presented in the study are included in the article, further inquiries can be directed to the corresponding author.

## Ethics Statement

The studies involving human participants were reviewed and approved by Medical Ethics Committee of the First Affiliated Hospital of Nanchang University. The patients/participants provided their written informed consent to participate in this study.

## Author Contributions

YS guarantees the integrity of the whole learning and the approval of the final version of the manuscript. R-BL is responsible for learning concepts, research design, and manuscript draft. L-QL is responsible for literature research, data analysis/interpretation. W-QS and TS are responsible for clinical experimental research, data collection, and statistical analysis. Q-MG, Q-YL, H-YS, and L-JZ are responsible for manuscript editing and original revision/review. All authors contributed to the article and approved the submitted version.

## Conflict of Interest

The authors declare that the research was conducted in the absence of any commercial or financial relationships that could be construed as a potential conflict of interest.

## Publisher’s Note

All claims expressed in this article are solely those of the authors and do not necessarily represent those of their affiliated organizations, or those of the publisher, the editors and the reviewers. Any product that may be evaluated in this article, or claim that may be made by its manufacturer, is not guaranteed or endorsed by the publisher.
